# Progranulin and Activin A Concentrations are Elevated in Serum from Patients with Acute Exacerbations of Idiopathic Pulmonary Fibrosis

**DOI:** 10.1007/s00408-021-00470-6

**Published:** 2021-08-30

**Authors:** Tian Xie, Lizhen Han, Yongxing Chen, Haihong Wu

**Affiliations:** 1 Department of Pulmonary and Critical Care Medicine, Hainan General Hospital, Hainan Affiliated Hospital of Hainan Medical University, 19 Xiuhua Road, Xiuying District, Haikou, 570311 Hainan Province China; 2 Department of Medical Records and Statistics, Hainan General Hospital, Hainan Affiliated Hospital of Hainan Medical University, Haikou, 570311 Hainan China

**Keywords:** Idiopathic pulmonary fibrosis, Acute exacerbation, Progranulin, Activin A

## Abstract

**Purpose:**

Idiopathic pulmonary fibrosis (IPF) is a chronic, progressive fibrotic lung disease of unknown cause with a variable course. Acute exacerbations of IPF (AE-IPF) is sudden accelerations of the disease or a superimposed idiopathic acute injury significantly reducing lung function. To examine the serum concentrations of Progranulin (PGRN) and activin A in patients with AE-IPF in a pilot study.

**Methods:**

Twenty-one patients with AE-IPF were compared with 23 patients with stable IPF as a control group. Serum PGRN and activin A levels, arterial blood gas measurements, and lung function were determined in these two groups.

**Results:**

Peripheral blood PGRN and activin A levels in patients with AE-IPF were 83.7 + 10.0 and 14.2 ± 1.7 ng/ml (mean + SD), respectively; higher than those in the control group 61.0 + 5.8 and 5.8 + 1.0 (*p* < 0.001). PGRN and activin A levels were significantly negatively correlated with carbon monoxide diffusion capacity *r* = − 0.857 (*p* < 0.001) and *r* = − 0.757 (*p* < 0.001).

**Conclusion:**

Progranulin (PGRN) and activin A may be involved in the pathogenesis of AE-IPF. They may be possible markers of disease activity in AE-IPF.

## Introduction

Idiopathic pulmonary fibrosis (IPF) is a chronic, progressive, and fibrotic interstitial pneumonia, the pathogenesis of which is not fully understood. There are currently no very effective therapeutic interventions other than lung transplantation, although pirfenidone and nintedanib are thought to slow deterioration of lung function and may reduce the frequency of acute exacerbations to some extent. The overall prognosis remains poor [1]. Most patients experience progressive decline over time but the clinical course can be highly variable [2]. Acute exacerbations of IPF represents sudden deteriorations in lung function occurring due to sudden accelerations in lung disease or superimposed acute lung injury which may be idiopathic.

Progranulin (PGRN) is a secreted 539-amino acid multifunctional growth factor thought to be involved in a variety of physiological and disease processes including embryogenesis, wound healing, inflammation, tumorigenesis, and host defense. PGRN is widely expressed in immune cells such as epithelial cells, macrophages, dendritic cells, and also within neurones. It plays a pro-inflammatory role in post-injury repair, in diabetes in the presence of insulin resistance, and in obesity and an anti-inflammatory role in lipopolysaccharide-induced acute pneumonia, in acute cerebral ischemia, and in various autoimmune diseases in mice [3]. There is evidence of its involvement in wound healing and diverse conditions such as psoriasis and colitis in humans and in mouse models [4]. In a recent study serum PGRN was significantly higher in dermatomyositis (DM) patients than in polymyositis or healthy controls. Concentrations were significantly raised in DM combined with interstitial lung disease and may be associated with reduced 6-month survival in this group [5]. Activin A is a multipotent cytokine, a member of the transforming growth factor beta (TGF-β) superfamily, which is involved in inflammatory, tumor, and fibrotic processes in a variety of tissues and organs and its expression is elevated in idiopathic pulmonary fibrosis [6].

We have found no studies on PGRN or activin A in acute exacerbations of idiopathic pulmonary fibrosis, and this study aimed to examine the concentrations of PGRN and activin A in the serum of patients with acute exacerbations and compare them with those in stable idiopathic pulmonary fibrosis to investigate any potential pathogenetic significance. The possibility that one or both proteins might have potential as clinical markers of AE-IPF was also considered.

## Materials and Methods

### General Data

Patients were selected from those who were hospitalized in Hainan General Hospital for AE-IPF between January 2017 and June 2020. All met the diagnostic criteria for AE-IPF [1]: (1) Previous or concurrent diagnosis of IPF, (2) Acute worsening or development of dyspnea typically of < 1-month duration, (3) High-resolution computed tomography (HRCT) with new, bilateral, ground-glass opacification, and/or consolidation superimposed on a background pattern consistent with usual interstitial pneumonia (UIP) pattern, and (4) Deterioration not fully explained by clinically significant cardiac failure or fluid overload or pulmonary embolism. The control group included patients with stable IPF who regularly attended follow-up in the Respiratory Medicine Outpatient Clinic of Hainan General Hospital. We applied the following criteria of IPF [7]: (1) Compatible clinical features, (2) The presence of a UIP pattern on HRCT (performed according to the recommended technical requirements): subpleural, basal predominance, reticular abnormality, honeycombing with or without traction bronchiectasis, and absence of features listed as inconsistent with UIP, (3) Exclusion of other known causes of interstitial lung disease including negative connective tissue serology, and (4) Agreement of the diagnosis by all members in a multidisciplinary discussion among respiratory physicians, imaging physicians, and pathologists with rich experience in the diagnosis of ILD (which was accepted by all of the patients). None of the patients underwent surgical lung biopsy. Exclusion criteria included IPF patients with concomitant: (1) chronic obstructive pulmonary disease (COPD), asthma, bronchiectasis, pulmonary infection, pulmonary embolism, or other respiratory diseases, (2) malignancy, (3) stroke, Alzheimer’s disease, acute coronary syndrome, type 2 diabetes mellitus, and obesity (BMI ≥ 30 kg/m^2^), (4) renal or hepatic dysfunction, cirrhosis, and fibrosis, (5) autoimmune diseases such as psoriasis, dermatomyositis, or rheumatoid arthritis, (6) patients receiving, or who had received, immunosuppressant or glucocorticoid therapy within the previous 3 months, and (7) patients receiving, or who had received, bronchodilators within the previous 3 months.

The experimental protocol was approved by the Human Ethics Committee of Hainan General Hospital. Written informed consent was obtained from individual participants.

Twenty-one patients were included in the AE-IPF study group and 23 patients with stable IPF were enrolled as a control group.

### Methods

Five milliliters of peripheral venous blood was drawn from fasting patients in both groups within 24 h after enrollment. Supernatants were extracted by centrifugation at 3000 rpm for 10 min. Serum levels of PGRN and activin A were measured using enzyme-linked immunoassay (ELISA) kits (R&D Systems, USA) according to the product’s specification. According to the kit specification, we diluted the samples before testing. Arterial blood gas analysis (Blood gas analyzer Radiometer ABL80) was performed immediately after 2 ml sample of peripheral arterial blood was collected. Pulmonary function tests (Medisoft Micro 5000, Belgium) were performed within 48 h after enrollment. The main variables recorded were the measured/expected ratio of Forced Vital Capacity (FVC) and diffusion capacity for carbon monoxide (DLCO) both expressed as percent predicted. Spirometry was conducted by the same physician according to the international guidelines, using a PFT spirometer (Medisoft Micro 5000, Belgium).

### Statistical Analysis

SPSS17.0 statistical software was used to analyze the patients’ data. The Kolmogorov–Smirnov test was performed on continuous data. Patients’ age, PGRN and activin A concentrations, blood gas measurements, FVC, and DLCO were presented as mean ± standard deviation (SD). The differences between groups were analyzed by Student's t test. Discrete and categorical data such as patient gender ratio and use of anti-fibrotic treatment were analyzed by χ2 test. Correlations between PGRN and activin A levels and DL_CO_ in all patients were analyzed by Spearman correlation analysis. All tests were two-tailed. *p* < 0.05 was taken as indicating a statistically significant difference.

## Results

The study group included 21 patients, 19 males and 2 females, aged between 52 and 71 with a mean age of 65.2 ± 6.8 years. The control group included 23 patients, 20 males and 3 females, aged between 49 and 68, with a mean age of 62.4 ± 5.7 years.

All patients in the study group had mild AE-IPF but all patients in the study required supplemental oxygen therapy admission to hospital. There were no statistically significant differences in general characteristics such as age, gender, duration of IPF, and baseline anti-fibrotic treatment status between the two groups, see Table 1 for details (*p* > 0.05).

**Table 1:** General characteristics of the enrolled patients

	AE-IPF	Control group	*χ*^2^/*t*	*p*-value
Number of patients	21	23		
Sex (male)	19 (90.5%)	20 (87.0%)	*χ*^2^ = 0.012	0.914
Age (years), mean ± SD	65.2 ± 6.8	62.4 ± 5.7	*t* = 1.485	0.145
Duration of IPF (years), mean ± SD	1.2 ± 0.3	1.0 ± 0.5	*t* = 1.589	0.119
Baseline anti-fibrotic therapy^a^	9 (42.86%)	12 (52.17%)	*χ*^2^ = 0.100	0.752

^a^Baseline anti-fibrotic therapy included pirfenidone and nintedanib

**Fig. 1:**
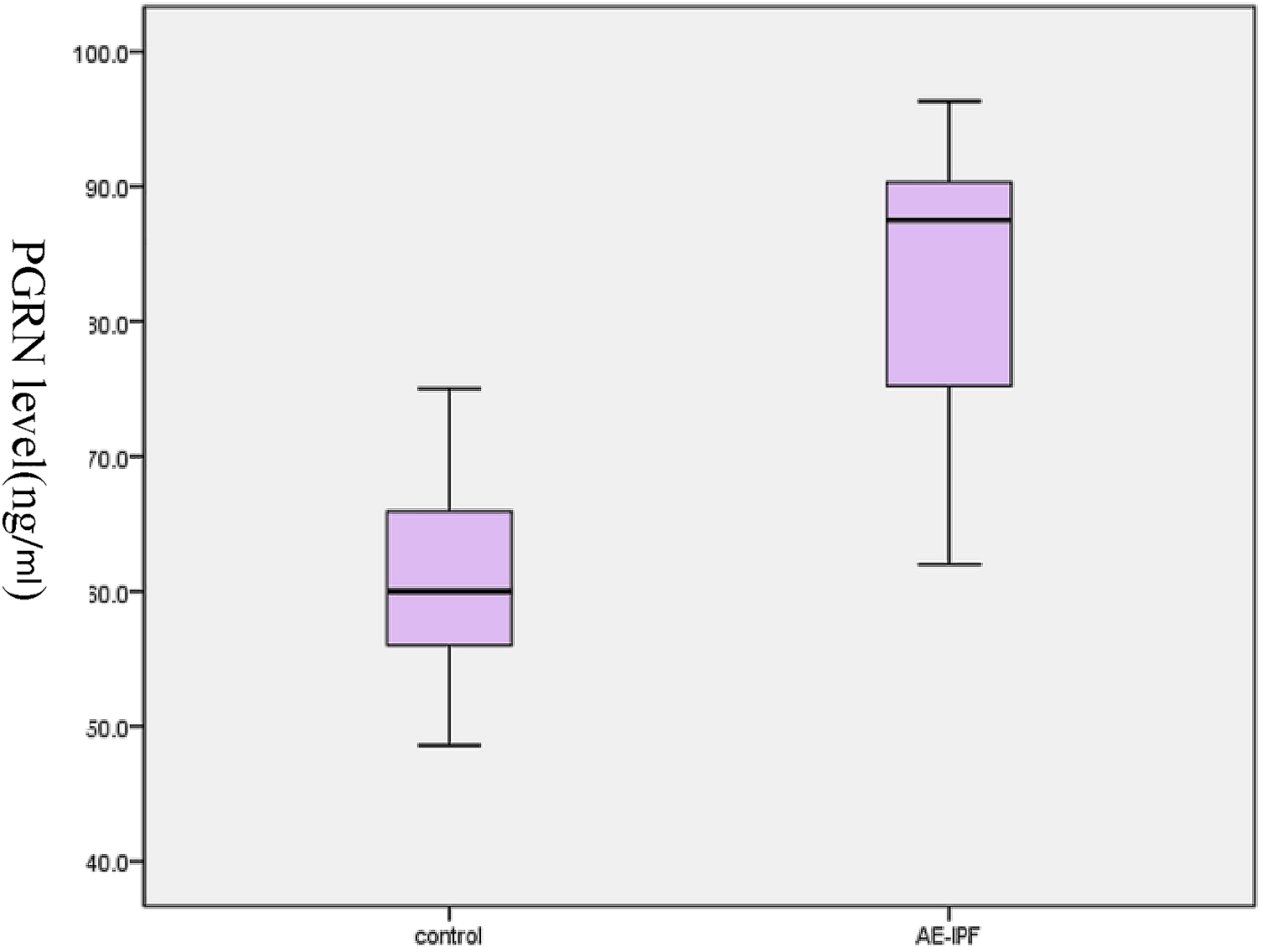
Plasma PGRN levels in patients with stable IPF and AE-IPF

**Fig. 2:**
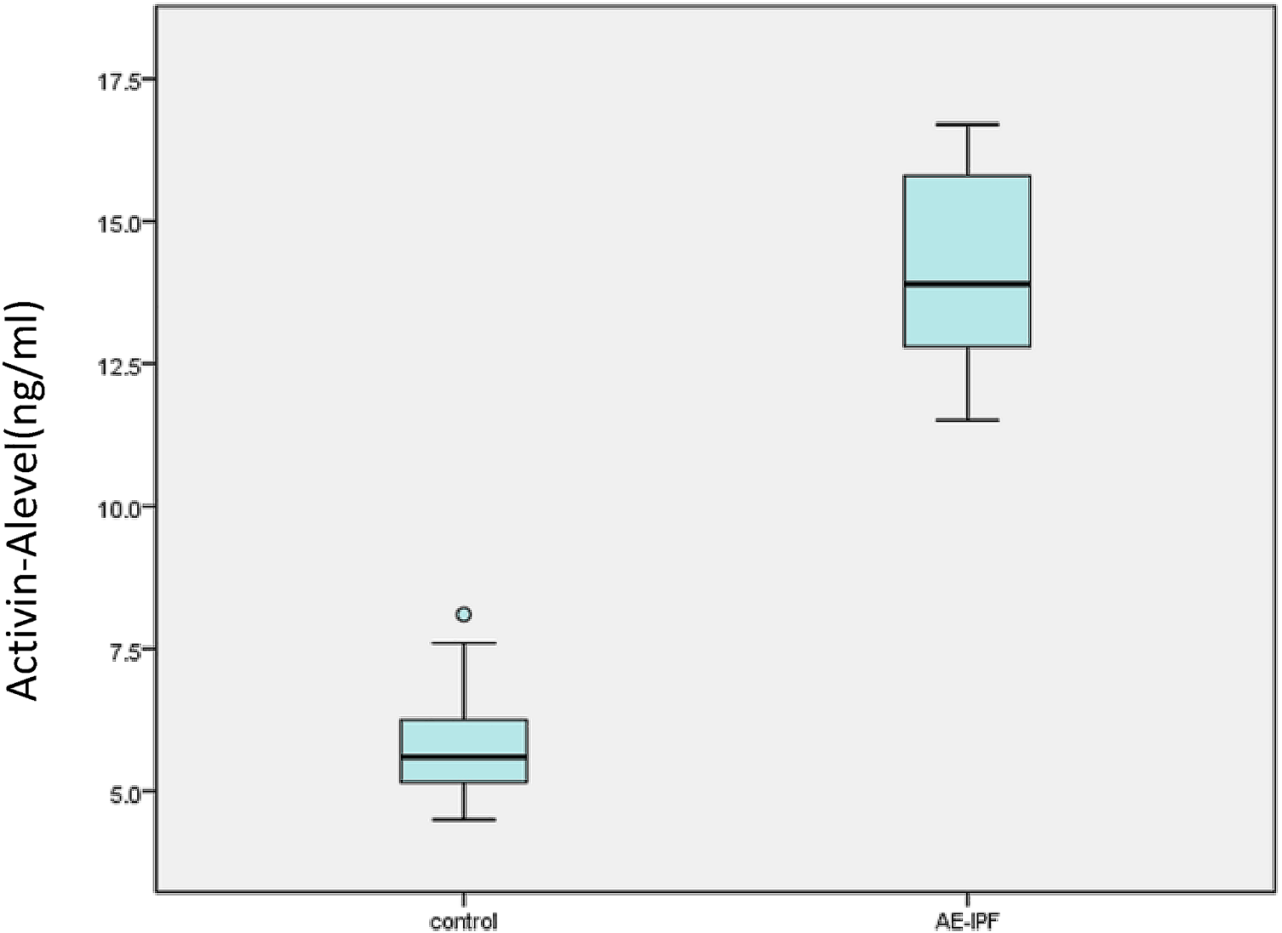
Plasma activin A levels in patients with stable IPF and AE-IPF

Mean FVC and DL_CO_ measurements were significantly lower in the study group compared to patients with stable IPF (*p* < 0.05, see Table 2 for details). The PGRN and activin A concentration data were normally distributed by Kolmogorov–Smirnov test. The serum levels of PGRN and activin A in the study group were significantly higher than those in the control group (*p* < 0.05) (see Table 2, Figs. 1, 2). The level of A-a gradient of oxygen in the study group was significantly higher than that in the control group (*p* < 0.05).

**Table 2:** Comparison of serum levels of PGRN, activin A, A-a gradient, DL_CO_% pred, and FVC% pred in the two groups

	AE-IPF	Control group	*t*	*p*-value
PGRN (ng/ml), mean ± SD	83.7 ± 10.0	61.0 ± 7.3	8.64	< 0.001
Activin A (ng/ml), mean ± SD	14.2 ± 1.7	5.8 ± 1.0	19.7	< 0.001
A-a gradient (mmHg), mean ± SD	38.2 ± 5.28	34.7 ± 3.61	2.58	0.013
DLco% pred, mean ± SD	33.8 ± 5.5	50.6 ± 5.4	− 6.79	< 0.001
FVC% pred, mean ± SD	39.4 ± 5.8	48 ± 6.0	− 4.566	< 0.001

A-a gradient: the difference in blood oxygen partial pressure between alveoli and arterial capillaries

DLco: carbon monoxide diffusion capacity % pred: percentage predicted

FVC: forced vital capacity % pred: percentage predicted

**Fig. 3:**
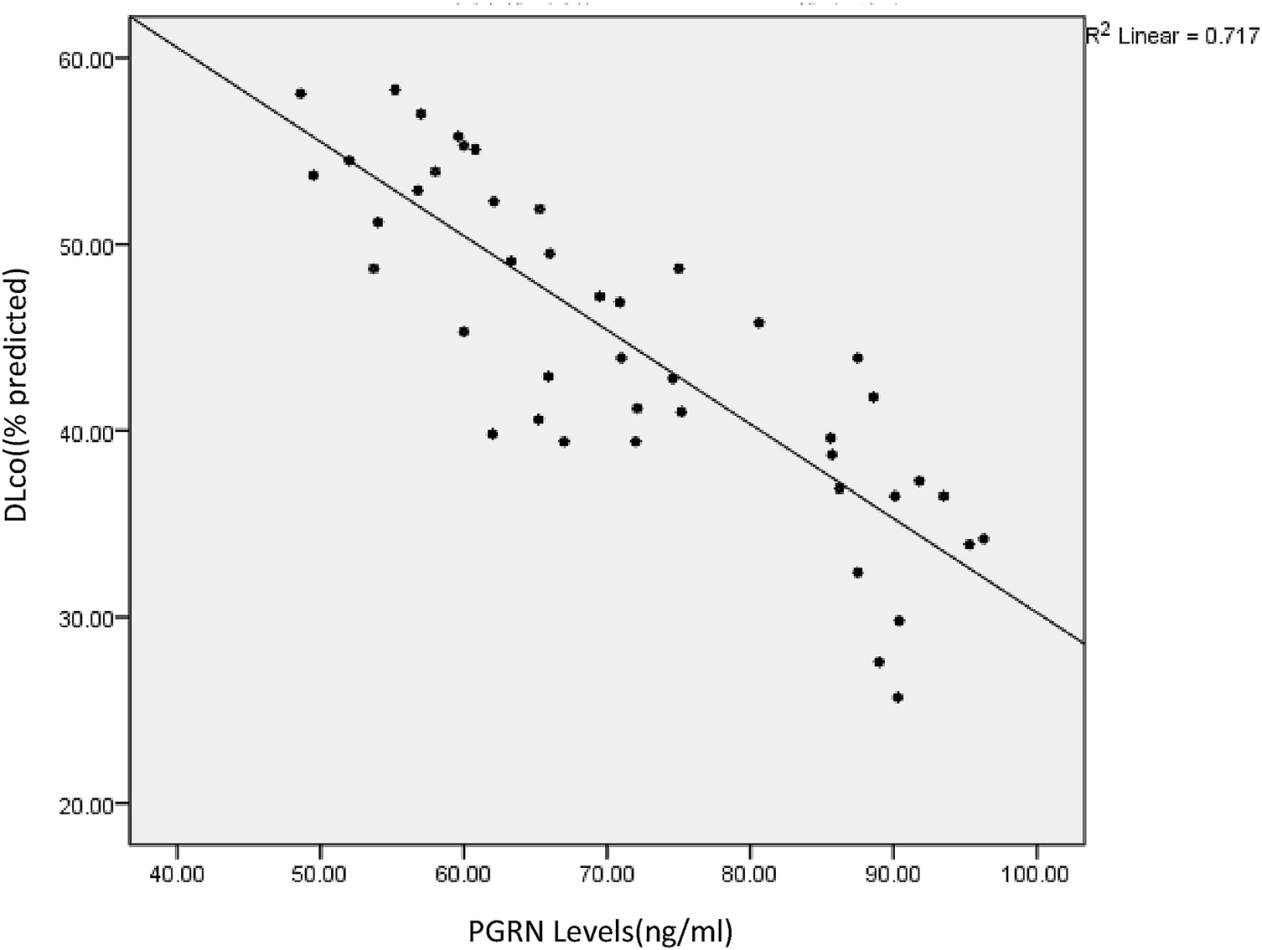
Correlation between PGRN levels and DLco; significant negative correlation between PGRN and DLco in IPF patients

**Fig. 4:**
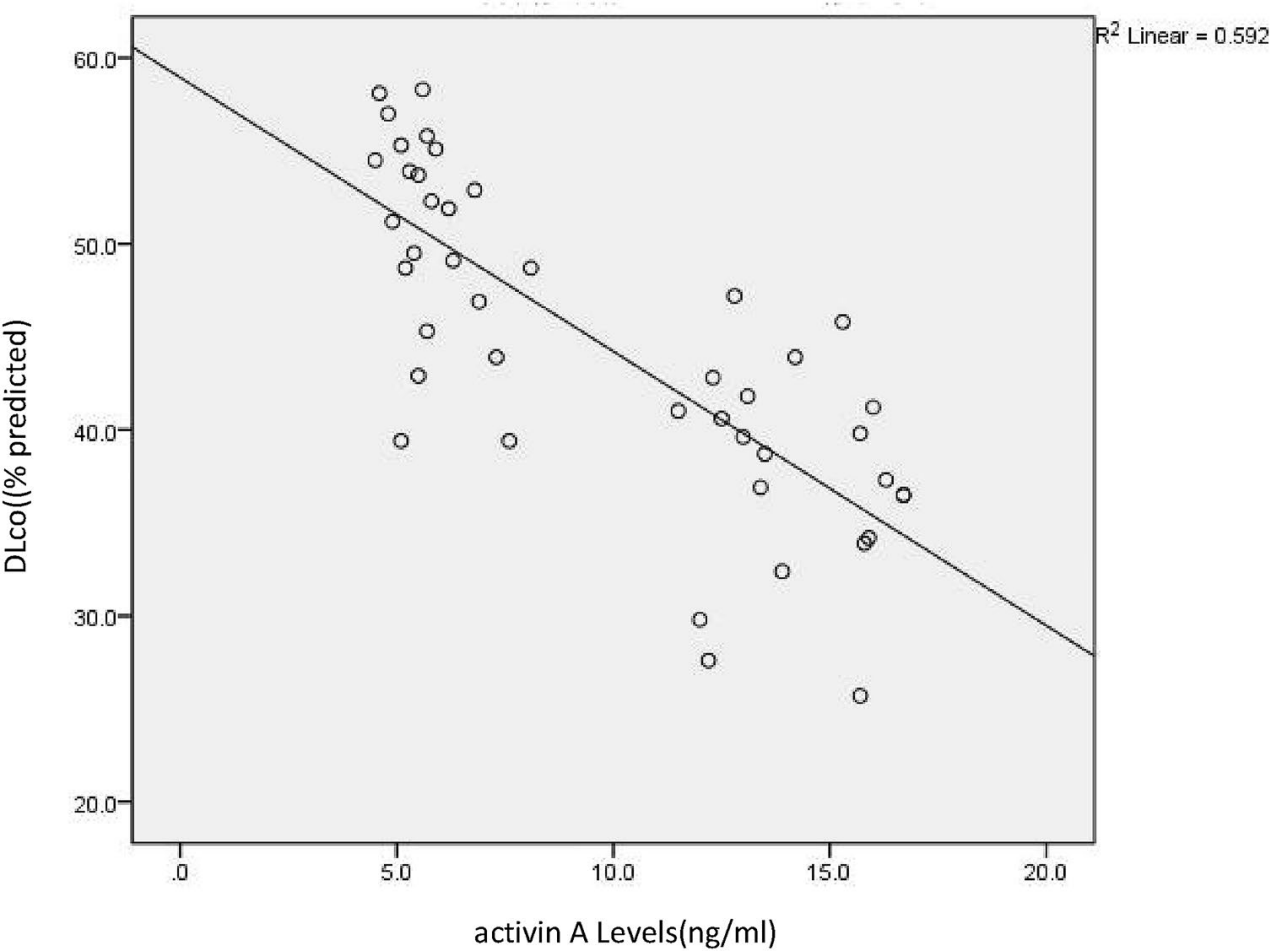
Correlation between activin A levels and DLco; significant negative correlation between activin A and DLco in IPF patients

A significant negative correlation was demonstrated between PGRN and DL_CO_; Spearman correlation coefficient *r* =  − 0.859, *p* < 0.001 indicating that serum PGRN concentration increased as DL_CO_ decreased (see Fig. 3). A significant negative correlation was also demonstrated between activin A and DL_CO_; Spearman correlation coefficient *r* =  − 0.757, *p* < 0.001 indicating that serum activin A concentration increased as DL_CO_ decreased (see Fig. 4).

## Discussion

Idiopathic pulmonary fibrosis is the commonest form of idiopathic interstitial pneumonia, the pathogenesis of which is not understood, although epithelial cell injury is thought to underlie abnormal healing and subsequent fibrosis. The natural history is variable; a steady predictable decline is common but some patients experience acute deteriorations of unknown etiology termed AE-IPF. This small, single-center study has shown significant elevations of PGRN and activin A in patients with mild AE-IPF compared with stable IPF. No a priori power calculation was possible to determine sample size as no previous estimates of PGRN or activin A were available in this population but this was intended as a pilot study. The PGRN concentrations determined in this study were similar to those measured by Tanaka et al. in DM with ILD [5]. The negative correlations of activin A and PGRN with DL_CO_ are of interest, although preliminary, as they could represent only linear interpolation of data from two distinct patient populations. However, if real, they may imply some cause and effect relationship; that is, whatever processes reflected by increase in these growth factors, bearing in mind their multiple possible cellular origins and their multiplicity of effects, may relate directly to acute deterioration in the diffuse alveolitis and disorganization of alveolar structures, ultimately leading to an acute exacerbation of the interstitial fibrosis. The negative correlation of PGRN and activin A with gas transfer but not with FVC is unexplained but, if genuine, could be because the changes in AE-ILD are mild reflecting inflammation or altered ventilation perfusion rather than factors affecting volume loss, e.g., fibrosis. The mechanism may be that PGRN and activin A are involved in the inflammatory response to common triggers of acute exacerbations of idiopathic pulmonary fibrosis such as bacterial or viral infections, gastric contents aspiration, and air pollution. Even if not mechanistically important PGRN and activin A could potentially be useful clinically as markers of AE-IPF.

Sputum PGRN concentrations have been reported to be higher in acute exacerbations of COPD (AECOPD) than in stable COPD patients or controls and to be negatively correlated with FEV_1_. It has been suggested that PGRN might be an independent predictor of AECOPD [8]. However, in asthma, including occupational asthma, serum levels were reduced compared to controls [9, 10]. Serum PGRN concentrations were reportedly raised in acute community acquired pneumonia and correlated with poor prognosis [11]. In mouse models of cigarette smoke-induced epithelial injury [12], endotoxin shock [13], and an LPS-induced acute lung injury model [14], PGRN depletion (knockdown) increases inflammation while over-expression or pre-treatment reduces it.

Activin A, a member of the transforming growth factor β family, is importantly involved in angiotensin II-mediated atrial fibrosis and in promoting fibroblast differentiation of endometrial mesenchymal stem cells [15, 16]. It participates in fibrosis in liver, pancreas, and kidneys [17]. Activin A can stimulate fibroblast differentiation into myofibroblasts inducing the proliferation of lung fibroblasts, airway smooth muscle cells, and bronchial epithelial cells [18]. Levels were raised in airways severe asthma [19] and in the serum of patients with COPD compared to healthy controls [20]. Activin A and activin B are expressed at increased levels in alveolar epithelial cells and inflammatory cells in patients with idiopathic pulmonary fibrosis [6], and the activin A antagonist follicle suppressor can also reduce bleomycin-induced pulmonary fibrosis [17].

Potential weaknesses of this study are the small sample size and the limited number of relevant parameters measured. Only patients with mild IPF-AE were examined. Factors related to IPF, but also possible co-morbidities, need to be further refined to exclude bias. Measurements were made only at a single time point and there is no sequential information about evolution of the signals. Future, larger, prospective, ideally longitudinal, studies will be needed to confirm these findings and to focus on trying to dissect possible pro-inflammatory and anti-inflammatory effects of PGRN and activin A and their mechanisms in IPF and particularly in AE-IPF. Comparison of PGRN and activin A with other possible markers of disease activity in IPF, e.g., KL-6 [21], Matrix metalloproteinases 1 and 7, and Surfactant Proteins A and D, chemokines, and cytokines such as chemokine ligand 18 and interleukin 8 [22] are urgently needed to assist diagnosis and treatment of acute exacerbations of idiopathic pulmonary fibrosis.

## Data Availability

All data and material generated or used during the study appear in the submitted article.
